# What Are the Effects of Short Video Storytelling in Delivering Blockchain-Credentialed Australian Beef Products to China?

**DOI:** 10.3390/foods10102403

**Published:** 2021-10-11

**Authors:** Shoufeng Cao, Marcus Foth, Warwick Powell, Jock McQueenie

**Affiliations:** QUT Design Lab, Queensland University of Technology, Brisbane, QLD 4000, Australia; m.foth@qut.edu.au (M.F.); wp@beefledger.io (W.P.); jock.mcqueenie@hdr.qut.edu.au (J.M.)

**Keywords:** short video, digital storytelling, blockchain, Chinese consumers, Australian beef, food experiences, consumer trust, food communications

## Abstract

Short videos have become the most-liked medium for Chinese consumers to learn about a brand’s products or services. This paper assesses how short video storytelling shapes Chinese consumers’ perceptions towards blockchain-credentialed Australian beef and their willingness to pay (WTP). A controlled experiment with a one-minute short video was implemented in an online survey. Respondents in the treatment group watched the video before filling out the survey, whereas respondents in the control group did not. The paper analyses and compares the empirical results from local (*n* = 76) and foreign (*n* = 27) consumers. Results illustrate that the short video, as part of our food communications, positively shapes consumer perception towards meat quality, labelling and traceability trust of Australian beef but has only slight or even negative effects on WTP. This could be due to the short video offering consumers a sense of supply chain visibility but not delivering the right messages to meet their expectation of blockchain credentials. Furthermore, short video storytelling effects vary among consumers with different socio-economic characteristics. Our results posit that short video storytelling can be a useful tool in communicating blockchain-credentialed food products but require the design of a tailor-made storytelling experience for diverse consumers.

## 1. Introduction

Globally consumers are looking for authentic food products that are safe and healthy [1]. This is highly echoed in the Chinese market, where discerning consumers nowadays have more spending power, enjoy greater choice, and are more concerned about food authenticity and safety. In recent years there has been a new cycle of consumption and a diversification of food experiences in China, which means that more affluent consumers can afford not only quality products but also expect new shopping and consumption experiences [2] and demand healthy and sustainable food produce [3,4]. Further, the outbreak of the COVID-19 pandemic has not only drawn heightened attention to the precarity and unsustainability of food supply chains [5,6] but also forced more and more consumers to expect retailers and suppliers to share legible and reliable information to guarantee food safety [7]. On this basis, Chinese food consumers have been changing their behaviour and are particularly seeking authentic experiences in their purchase and consumption beyond simply ensuring food safety. In other words, Chinese consumers not only have a preference for safe and healthy products, but also are keen to know more about product-related information and the story behind the products themselves which is referred to as ‘food provenance’ [8]. However, food provenance communications with Chinese consumers are challenged by persistent food fraud incidents, such as adulteration, counterfeiting and misrepresentation of food products, which have disrupted the trust level among Chinese consumers [9].

Australian beef products are exposed to a higher counterfeiting risk in China [10]. To build consumer trust in the Chinese market, Australian beef industry bodies and exporters have launched various communication approaches with a view to enabling consumers not only to buy and consume true Australian beef products, but also to have more enjoyable experiences. For example, Meat & Livestock Australia has deployed the ‘True Aussie Beef’ label to give consumers a sense of authenticity. However, these static cling labels cannot deliver an appealing and trustworthy message to consumers. In response, several traceability systems have been launched to enable consumers to have easy and convenient access to a wealth of food product information by scanning a Quick Response (QR) code. Despite these efforts, consumers may still not be convinced whether they can trust this information. This is where blockchain technology that is described as a trusting machine comes in. Its immutable and tamper-proof characteristics in maintaining data integrity offer interesting possibilities for food product credentialing beyond its original and most prominent use as a cryptocurrency (e.g., Bitcoin) [10,11,12,13]. Various blockchain-based food traceability systems that offer track and trace functionality to verify food products by querying transaction or event updates recorded on a shared ledger have been developed [14,15] and the effects of blockchain on consumer perception have been measured [16]. However, existing research does not disclose much information about how blockchain works to deliver trustworthy information throughout the supply chain to consumers. Although consumers have heard a lot about blockchain-credentialed food products, they often have limited knowledge of what they are and blindly put their trust in them. This could give rise to the risk pertaining to customer value [17]. Therefore, it requires effective food communications with consumers in delivering food products that claim to be blockchain-credentialed.

Digital technology has significantly changed the way to access and consume information. It is reported that 72% of people prefer watching a video over texts to learn about a product or service [18]. Chinese consumers are keen on watching short videos to access information or know a new product or service. In this context, China’s short video industry is booming with 821 million users as of June 2019 [19], and businesses have reaped significant benefits from short video marketing on platforms such as Douyin (known as TikTok outside China). It is reported that in the first half of 2019 China’s e-commerce platform saw their sales conversion rates increase by 40% leveraged by short video marketing [20]. The rapid development of the short video industry in China offers the food export industry a novel approach to communicate with consumers. However, little is known about the value of short video storytelling on food products from a consumer perspective.

This paper fills these research gaps and examines the effects of short video storytelling in delivering blockchain-credentialed Australian beef products following an exploratory research approach. The study investigates the effects of a one-minute short video storytelling on consumer perceptions with respect to meat quality, trust in labelling and traceability data, and willingness to pay (WTP) using an in-field survey administered in control and treatment settings. The contribution of this study is two-fold. First, it offers an understanding of how a one-minute short video can shape Chinese consumers’ quality and trust perceptions towards blockchain-credentialed Australian beef products and its impact on WTP from a storytelling perspective. Second, it identifies those effects of short video storytelling vary among consumers with different socio-economic characteristics, which highlights the need for using diverse video strategies when targeting different groups of consumers.

Following the introduction section, Section 2 presents relevant literature. The study’s research methodology is discussed in Section 3, which is followed by our analysis of empirical data and key results in Section 4. The synthesis and discussion of results against current discourse in the literature is presented in Section 5 before the paper’s conclusions in Section 6.

## 2. Literature Review

We review relevant literature pertaining to digital storytelling and short videos (Section 2.1) and food consumer perceptions (Section 2.2).

### 2.1. Digital Storytelling

Digital storytelling refers to the way of using digital tools to tell stories, and store and exchange those stories on websites and social media networks in the Internet era [21,22]. The development of digital tools, such as smartphones, location technologies and multimedia content platforms has greatly facilitated the production and distribution of digital storytelling [21,23,24]. Prior research has found that digital storytelling has become a powerful way of interpreting additional information [25] and can therefore influence people’s perception and intention [26]. As such, digital storytelling has been used in many areas, including education [27,28], tourism [26,29,30], science communication [31], community engagement and digital inclusion [32] and technology acceptance [33]. Surprisingly, to our best knowledge, there is still little understanding of how digital storytelling influences consumer perceptions and consumer behaviours.

Digital technology has changed the ways of creating and consuming narrative stories [34,35]. Although digital storytelling still processes traditional storytelling methods, new multimedia tools have equipped storytelling with video or animation configuration [36,37]. The role of video digital storytelling has attracted interest from scholars. For example, Pera and Viglia [38] found that video digital storytelling enables rational, emotional and relationship experiences in online peer-to-peer communities. More recently, short video storytelling has been flourishing in China owing to the rise of short video sharing mobile applications like Douyin and Kuaishou [39]. Short videos with a clip duration of one minute or less [40] have rapidly become a social video-marketing genre in addition to traditional texts or audio-visual content [41]. Due to the recent rise of short videos as a new form of video digital storytelling in China, there is scarce empirical research reporting the value of short videos. This paper starts to fill this research gap by exploring the use of short videos as a new form of digital storytelling in influencing consumer perception towards food quality, label trust and traceability and willingness to pay (WTP) among Chinese consumers.

### 2.2. Consumer Perception

Consumer perception refers to an information process involving consumer exposure and attention to marketing stimuli and consumer interpretation to create a meaningful image and brand identity [42,43]. While consumer perception is a complex and dynamic process, the Stimulus–Organism–Response (S-O-R) theory can explain how consumer perception is influenced and how it affects other factors, such as consumer attitude, purchase decision and willingness to pay (WTP). The S-O-R theory is based on environmental psychology that states that people’s behavioural responses are the result of the stimuli from the environment and their internal emotional and cognitive states [44]. The S-O-R theory posits that an individual’s response is driven by their exposure to stimuli from the environment, which has proven useful in consumer behaviour studies [45,46,47]. Built on the S-O-R theory, this paper assumes that consumer perception towards meat quality, label trust and food traceability is influenced by the information/content they receive, which in turn affects their purchase decisions and willingness to pay (WTP).

A growing number of studies have explored customer perception of food products in terms of quality, label, traceability, authenticity and so on [48,49,50,51,52,53,54,55,56]. Early research has found that consumer perception can affect buying decisions [57] and lead to changing consumer behaviour [53,55]. As such, the meat industry regards consumer perception of meat and meat products as a critical issue to improve consumer trust, sales and profitability [53]. Accordingly, there is growing interest in consumer perception of food quality [48,49,50]. Some studies have offered insights into how consumer perception affects quality, label trust and willingness to pay (WTP). For example, Udomkun et al. [58] reported a significant effect of consumers’ perception on purchasing decisions and WTP for meat products. Suhandoko et al. [59] investigated consumer perception and WTP for information attached with pork traceability in Taiwan’s traditional markets. To our best knowledge, there is limited understanding of the effect of digital storytelling on consumer perceptions and behaviours in the food industry. Given that short videos have become one of the favourite ways of receiving information among new generations of consumers [60], this paper presents empirical results from an exploratory study of how short video digital storytelling affects consumers’ perception and WTP.

## 3. Methodology

### 3.1. Research Experiment

The effects of how short video storytelling shapes consumers’ perceptions towards food quality, label trust and traceability of blockchain-credentialed Australian beef and affects their willingness to pay (WTP) were measured with a controlled experiment. The controlled experiment is exploratory in nature, consisting of a one-minute short video and six questions (Table 1) that comprised the second part of a larger survey with 16 questions in total. The survey was delivered to explore consumers’ attitudes and expectations about blockchain-credentialed Australian beef products in China.

**Figure 1:**
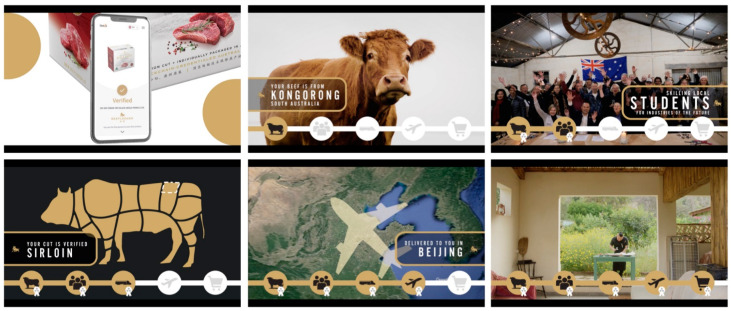
Six screenshots of the one-minute video showing from top left to bottom right: QR code scanning feature; visuals of food provenance and the region of origin in South Australia; visuals of the producer communities including high school students; illustration of the relevant cuts of beef; track and trace of the product’s routing from Australia to China; visuals of food preparation by consumers in China. Source: Authors. For web links to video see Appendix A.

The one-minute video -illustrated the use of blockchain to verify key stages from paddock to plate and introduced people and stakeholders from local food production communities in regional South Australia and other supply chain participants. Figure 1 shows a collage of six video screenshots. The rationale for measuring the effects of the short video is grounded in the fact that over recent years Chinese consumers have started to prefer short videos over texts or messages in receiving product-related information [60].

**Figure 2:**
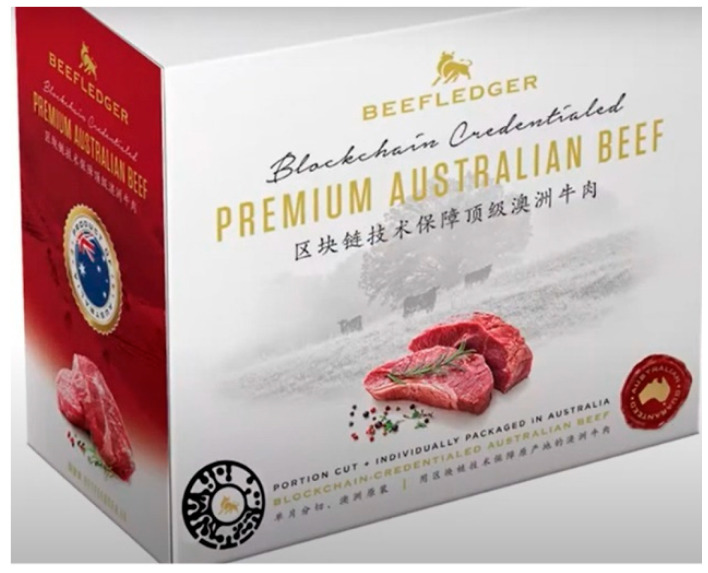
Beef package with a traceable fingerprint. Source: authors.

To ascertain food quality perception, the study examined whether Chinese consumers perceive Australian beef products cut and packed in Australia to be superior to those processed in China. Consumer trust in food labelling was measured by examining consumers’ level of certainty in the food provenance of the beef labelled ‘Australian cut and packed.’ The traceability trust refers to the level of consumer trust in the traceability information attached with the beef package via a digital fingerprint (Figure 2). The WTP was measured with an anchor price of 150 g of Australian portion cut and packaged Sirloin steak that was set at 90 Yuan (RMB) with reference to the retail price from an online retail platform JD.com. A consumer’s intention to pay extra was measured by three categorical variables ‘yes,’ ‘no’ and ‘not sure.’ The ‘WTP more’ variable was captured by asking how much more the consumer was willing to pay.

Respondents were randomly assigned into either the control or the treatment group to answer the questions illustrated in Table 1. Respondents assigned to the treatment group watched the one-minute video before answering survey questions, while those in the control group did not watch the video.

### 3.2. Data Collection

The survey was conducted during a series of showcase dinner events across China’s four metropolitan cities Shanghai, Shenzhen, Beijing and Guangzhou from 18 to 25 November 2019. The survey was administered using an online survey platform (*wj.qq.com*) that allows the delivery via *WeChat to* invited event attendees.

Table 2 reports the demographic characteristics of 103 valid samples, which is reasonably accepted in an exploratory study designed within control and treatment settings [61]. The control and treatment groups comprise 48 and 28 local respondents and 14 and 13 foreign respondents respectively. Most local and all foreign respondents in the control and treatment groups were from the four metropolitan cities. Middle-aged respondents accounted for the largest portion in all groups (the foreign treatment group excluded). Over 60% local and foreign respondents in both the control and treatment groups have college or equivalent qualifications, bachelor’s degree, or master’s degree. Only the local treatment group comprised more female respondents (54.2%). All group respondents, excluding the foreign control respondents, were scattered across the five household income groups wealthy, upper, middle, and low-income that were based on the 2018 classification of household income in China, and over two-thirds local and foreign respondents in both control and treatment groups came from the wealthy and upper groups.

### 3.3. Data Analysis

A descriptive analysis was initially conducted to illustrate the distribution of the data and identify associations among variables. Mann–Whitney *U* tests were then conducted to compare the responses of those who watched the video to those who did not. The Mann–Whitney *U* test is a nonparametric test that determines whether two independent groups were drawn from the same population that are not normally distributed [62]. The null hypothesis is that there is no difference between the responses for one group compared to the other. The null hypothesis is rejected if *p*-value for the t-test is less than 0.05. The effects of socio-demographic variables, including city of residence, age (see note in Appendix B), education, gender and marriage, and annual household income, on responses from local and foreign respondents were assessed with one-way ANOVA tests. ANOVA is a statistical technique that assesses potential differences in a scale-level dependent variable by a nominal-level variable having two or more categories [63]. The null hypothesis for an ANOVA is that there is no significant difference among the groups. If the null hypothesis is rejected, it means that there is at least one significant difference among the groups.

## 4. Results

In this section we present our findings analysing the impact of the short video on food consumers by focussing first on trust and meat quality perception (Section 4.1) and then on willingness to pay (Section 4.2).

### 4.1. Short Video Storytelling on Trust and Meat Quality Perception

**Figure 3:**
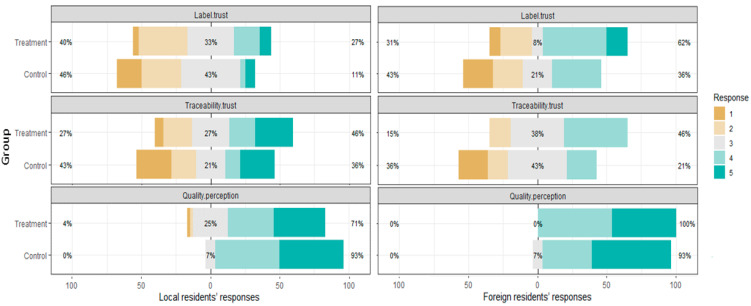
Comparison of quality perception and label and traceability trust by group. Source: Authors.

Figure 3 compares the responses from those who watched the video with those who did not with respect to their perceptions of trust in labelling, meat quality and traceability data. The intervention of the short video storytelling sees increased trust perceptions towards the label credence claims. 27% of local respondents and 62% of foreign respondents in the treatment groups trust the authenticity of the ‘Australian cut and packed beef’ label compared to 11% of local respondents and 36% of foreign respondents from the control groups. The increase of trust perception is also found in the product traceability information. 46% of local and foreign treatment respondents express traceability trust, compared with 36% of local respondents and 21% of foreign respondents in the control groups. Differently, local and foreign respondents have mixed meat quality perceptions with the short video intervention. 100% of foreign treatment respondents compared with 93% of their control counterparts state that Australian beef products cut and packed in Australia are better than those processed in China. Interestingly, 71% of local treatment respondents support the superiority of ‘Australian cut and packed,’ which is lower than their control counterparts (93%).

Table 3 reports the Mann–Whitney *U* test for respondent perceptions between groups. No significant differences (*p*-values ≥ 0.05) with respect to trust and meat quality perceptions between the control and treatment groups are found at a 95% confidence level for both local and foreign respondents. In other words, while our short video storytelling increases consumer perceptions towards meat quality, label trust and traceability trust, it did not yield significantly comparative differences.

Table 4 presents results of the one-way ANOVA tests for the effects of socio-demographic variables on trust and meat perceptions for treatment respondents. The effects of education on meat quality and city of residence on traceability trust are statistically significant at a confidence level of 95% and the effects of city of residence on label trust is statistically significant at a confidence level of 90% for local treatment respondents. In contrast, there is a significant difference found in the effects of age on label trust at a confidence level of 90% for foreign treatment respondents.

### 4.2. Short Video Storytelling on Willingness to Pay

**Figure 4:**
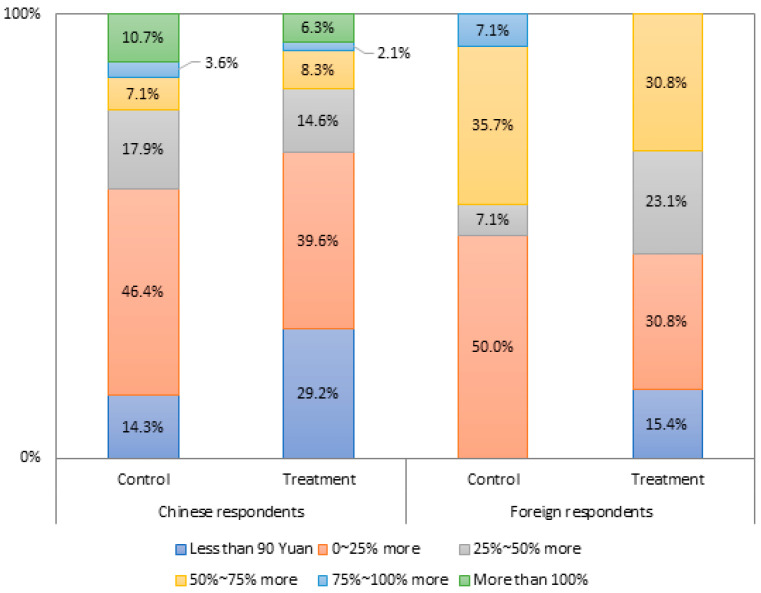
Percentage distribution on WTP prices against the anchor price by group. Source: Authors.

Figure 4 shows the percentage distribution of WTP for a 150 g Blockchain-credentialed Australian cut and packed sirloin steak between the control and treatment groups. Most local and foreign respondents are willing to pay more than the anchor price (approx. 90 yuan), with the price premiums up to 25% above the reference price taking the largest portion. Surprisingly, the study’s short video storytelling intervention yields negative results. 70.83% of local treatment respondents and 84.62% of foreign treatment respondents are willing to pay more than the reference price, compared with 85.71% of local respondents and 100% of foreign respondents in the control groups. Higher means of WTP were also found in the control groups compared with the treatment groups. The mean WTPs from local and foreign respondents in the control groups (123.04 and 127.86 Yuan) are higher than those in the treatment groups (104.58 and 117.31 Yuan).

Table 5 compares consumers’ willingness to pay more between local and foreign respondents in the control and treatment groups. Similarly, the short video storytelling intervention sees a lower intention of consumers to pay more from the treatment respondents. 64.58% of local treatment respondents and 84.62% of foreign treatment respondents intend to pay more, compared with 78.57% of local control respondents and 92.86% of foreign control respondents. Local and foreign respondents in the control and treatment groups have varying degrees of being willing to pay more. Local treatment respondents are willing to pay more, averaged at 44.71 Yuan compared with their control respondents at 31.57 Yuan. In contrast, foreign treatment respondents are not willing to pay more than their control counterparts, with respective mean values at 52.00 and 57.31 Yuan.

Table 6 shows the Mann–Whitney *U* test for WTP variables between groups. No significant differences (*p*-values ≥ 0.05) for WTP between the control and treatment groups were observed at a 95% confidence level for both local and foreign respondents. This means that our short video intervention did not yield significant differences.

Table 7 presents results of the one-way ANOVA tests for the effects of socio-demographic variables on WTP variables for those respondents who watched the short video. There are statistically significant differences in the effects of gender and marital status on WTP at a confidence level of 90%, and respondents’ city of residence on their willingness to pay more at a confidence level of 95% for local treatment respondents. In contrast, there is a significant difference found in the effects of education on willingness to pay more at a confidence level of 95% for foreign treatment respondents.

## 5. Discussion and Implications

### 5.1. Discussion of Key Findings

Little is known about the effect of digital storytelling on consumer perceptions and behaviours in the food industry. Our results corroborate findings of previous studies that focuses on consumer perceptions and purchase decisions but also demonstrate new insights into the use of digital video storytelling in a new field. Comparing the responses from the control and treatment groups shows that our short video storytelling intervention increases consumer trust in the Australian ‘portion cut and packaged’ label and traceability information. This can be attributed to the improved transparency of the supply chain processes, which has been revealed in previous work [64]. Most local and foreign respondents who did not watch the video assume that Australian processed beef is superior to Chinese processed Australian beef. The reasons can be found in previous research that has revealed that the level of trust dropped when consumers found out that food was cut-up or re-packaged in China [65]. A decreased percentage of local treatment respondents assume the superiority of Australian processed beef products after watching the short video. This highlights that the use of short video storytelling to interpret blockchain credentials may lead to changes in consumer confidence and trust in Australian beef products processed in Australia and China.

Füzesi et al. [66] found a lack of effective education advertising and premium price of additional information are two barriers to consumer’s acceptance of blockchain-based traceability. Our papers measured consumer’s knowledge of blockchain-based traceability and examined their willingness to pay premium for blockchain credentialed beef products. The results show that both local and foreign treatment respondents are not willing to pay more for a 150 g blockchain-credentialed Australian cut and packed sirloin steak compared with their counterparts in the control group. This is probably because treatment respondents who watched the short video simply expect more from a digital video storytelling than their control counterparts who proposed their WTP prices based on their expectations only. The expectation gap of the short video storytelling in describing blockchain-credentialed beef products from the treatment respondents results in their lower WTP prices. This suggests it is essential to develop and deliver more tailor-made video storytelling experiences suited to a diversity of consumer profiles and expectations. The consumer’s willingness to pay more is found to be different between the control and treatment groups for local and foreign respondents, which is probably due to the varying attitude to the short video between local and foreign respondents.

Our research investigated consumer trust and perception research in a comparative setting, which differs from early research. The Mann–Whitney *U* tests for consumer trust and quality perceptions between the control and treatment groups show no significant differences at a 95% confidence level for both local and foreign respondents. Similarly, there are no significant differences at a 95% confidence level between treatments and controls in WTP price thresholds. These results show that while our current short video storytelling intervention increases consumer perceptions towards trust in food labelling and traceability data as well as quality, it is not powerful enough to yield significant differences when comparing results between the control and treatment groups.

Aligning with early consumer behaviour research in the food industry, our study explored if the effect of short video storytelling differs among consumers’ social-demographic characteristics. Our results contradict previous studies exploring consumers’ willingness to pay a premium for traceability certified beef [67] and consumers’ willingness to pay for organic food [68], which found education, marital status, gender, and age to be insignificant factors. One-way ANOVA results pertaining to the treatment responses to meat and trust perceptions indicate that education and city of residence are the most influential socio-demographic variables for meat quality perceptions for the study’s local respondents, and label and traceability trust, while respondent ages have significant effects on label trust for foreign respondents. One-way ANOVA results pertaining to the treatment responses to WTP variables reveal that gender and marital status and city of residence are most influential socio-demographic variables on WTP price thresholds for local respondents, while for foreign respondents it is education that has the greater effect on consumers’ willingness to pay more. Additionally, our results reveal that effects of short video storytelling could vary among consumers with different socio-economic characteristics, which highlights the need for adopting an approach to create diverse video storytelling experiences catered to different groups and socio-demographic profiles of consumers.

### 5.2. Theoretical Contribution

This study underpins the Stimulus-Organism-Response (S-O-R) theory from the perspective of a short video storytelling intervention. The S-O-R theory posits that an individual’s response is driven by their exposure to environmental stimuli [44] and therefore behaviour cannot exist without a stimulus. Several studies [45,46,47] have extended the S-O-R theory to investigate consumer behaviour in various domains. As opposed to these studies that used traditional texts and images, our study examines short video storytelling as a stimulus and examines how it shapes consumer perception towards food labelling and trust in traceability information as well as meat quality in the case of blockchain-credentialed beef products. Built on the assumption that consumer behaviours are influenced by the information/content they receive and consume, our study contributes to the understanding of food communications by examining consumer perceptions and WTP. Furthermore, this study contributes towards consumer education across the groups of population by showing the exploratory effects that short videos have on consumer perceptions and WPT.

### 5.3. Practical Contribution

This study also offers practical contributions to knowledge. Videos have become a powerful marketing tool, which is used by 86% businesses surveyed in 2021 [69]. According to Wyzowl’s survey, 93% of marketing professionals believe that video is an important part of their marketing strategies for consumer engagement and brand trust. Our study provides empirical evidence to support this statement. While our short video storytelling intervention did not yield significantly comparative impacts on consumer WTP but leading to increased consumer perceptions of meat quality and trust in labelling and traceability information. Corroborating similar findings by Kim and Hall [33], this demonstrates that digital video storytelling generally has a positive influence in the pursuit of marketing blockchain-credentialed Australian beef products. Our exploratory results can compel decision-makers in industry to strategically consider the positive effects that short video storytelling can have on consumer perceptions and purchase behaviours. Businesses thus need to consider including video content as part of their digital marketing strategy if they do not want to miss out on the opportunities that video content can offer.

Using short video storytelling to interpret blockchain credentials leads to changes in consumer confidence and trust in Australian beef products processed in Australia and China. This finding provides additional benefits to Australian businesses building China-based processing plants and Chinese food importers. Our ANOVA results reveal that short video storytelling effects vary among consumers according to different socio-economic characteristics. With this understanding, businesses should be aware of the importance of identifying how to deliver the right digital content information to the targeted population when they use short video for product storytelling. Furthermore, short videos can be an effective and entertaining tool to convey authentic food provenance stories and offer a new and emerging genre of consumer information in the complex and rapidly changing digital world, where consumers are already exposed to an overabundance of information in their everyday lives.

## 6. Conclusions

This present study implemented and tested a controlled experiment to investigate the effects of a short video storytelling intervention on consumer perceptions of meat quality, label, and traceability trust and on willingness to pay among consumers in China. By comparing the responses from local and foreign respondents in the control and treatment settings, our exploratory study revealed that while the short video storytelling intervention did not yield significant differences between the control and treatment groups, it did have a positive influence on consumer (both local and foreign) perception of quality and trust of Australian beef. Further, the effect of the short video storytelling intervention varies among consumers according to different socio-economic characteristics. The novelty of this study lies in offering an understanding of how short video storytelling can shape consumers’ quality and trust perceptions towards Blockchain-credentialed Australian beef and affect WTP from a short video storytelling perspective.

Although offering valuable early insights, this study has a few limitations that can prompt inspiration for further research. First, our study is exploratory in nature with a relatively small sample of 103 consumers situated in four metropolitan cities in China, which could lead to biased results. Accordingly, further research is required in order to produce findings generalisable to a broader context. Additionally, comparative research looking at potential differences in consumer perceptions depending on residency in different Chinese city tiers may deliver interesting results [70]. Second, questions pertaining to evaluating the control experiment were embedded in an online consumer survey. The online delivery reduces our control in managing how participants provide their answers to our designated questions, which results in an uneven number of respondents in the control and treatment groups. In order to unpack some of the results of this study, it would be worthwhile to conduct more nuanced, qualitative research to better understand diverse consumer experiences and reactions. Despite these two limitations, our exploratory study produced new insights that can pique the interest of both academics and readers in the food industry to contemplate how best to embed short video storytelling experiences in communicating and delivering food products to consumers.

Given the exploratory nature of our study and the abovementioned limitations, further studies are suggested to evaluate the role and impact of short video storytelling more comprehensively, with either more controlled experiments or qualitative research approaches. Measuring the effectiveness of short videos in comparison with other multimedia tools is also a promising research direction. As short videos can be presented with user-generated content (UGC) and professional-generated content (PGC), future research could also be tasked with providing new insights into the value of short video storytelling by comparing the effects of UGC and PGC. Finally, as consumer perceptions are not stable but rapidly evolving over time [50], future research is suggested to track and capture dynamic impacts of short video interventions.

## Figures and Tables

**Table 1:** Testing items and experiment question descriptions.

Testing Item	Question
Willingness to pay (WTP)	The retail price for a 150 g Sirloin steak claimed to be cut and packed in Australia is approximately 90 yuan. How much are you willing to pay for a BeefLedger branded Australian cut and packed Sirloin steak?__Yuan.
Label trust (1 = Not at all sure; 5 = Strongly sure)	When you buy a steak labelled Australian cut and packed beef, to what extent do you feel sure that the meat came from Australia?
Quality perception (1 = Totally disagree; 5 = Totally agree)	To what extent do you agree that Australian cut and packed beef products are superior to Chinese processed Australian beef products?
Traceability trust (1 = Not at all trustful; Extremely trustful)	To what extent do you trust the traceability information of Australian cut and packed steaks?
Intention to WTP more (Yes/Not sure/No)	Are you willing to pay extra for Australian cut and packed steaks with Blockchain credentialed traceability information?
WTP more	How much more are you willing to pay for a BeefLedger branded Australian cut and packed Sirloin steak with Blockchain credentialed traceability information: __Yuan.

Note: 100 Chinese Yuan = ~US$ 15.51 = ~€ 13.38 (as of 1 October 2021).

**Table 2:** Characteristics of the 103 local and foreign respondents.

Category	Items	Local Respondent (76)		Foreign Respondent (27)	
		Control (%)	Treatment (%)	Control (%)	Treatment (%)
City of residence	Beijing	42.9	27.1	7.1	30.8
	Guangzhou	17.9	18.8	21.4	23.1
	Shanghai	14.3	31.3	42.9	30.8
	Shenzhen	10.7	12.5	28.6	15.4
	Other	14.3	10.4	-	-
Age	18–25	10.7	25.0	-	15.4
	26–30	21.4	16.7	7.1	0.0
	31–40	25.0	18.8	42.9	0.0
	41–50	32.1	29.2	21.4	38.5
	51–60	7.1	8.3	14.3	30.8
	Over 60	3.6	2.1	14.3	15.4
Education	Middle school and below	3.6	-	-	-
	High school or equivalent	10.7	6.3	7.1	-
	College or equivalent	21.4	16.7	21.4	30.8
	Bachelor’s degree	32.1	41.7	35.7	46.2
	Master’s degree	28.6	33.3	35.7	23.1
	PhD and above	3.6	2.1	-	-
Gender and marital status	Female single	14.3	33.3	-	7.7
	Female married	25.0	20.8	7.1	15.4
	Male single	14.3	14.6	57.1	23.1
	Male married	42.9	31.3	35.7	46.2
	Other	3.6	-	-	7.7
Annual household income	30,000 Yuan and below	7.1	2.1	-	7.7
	30,000–80,000 Yuan	7.1	6.3	7.1	7.7
	80,000–150,000 Yuan	17.9	12.5	14.3	15.4
	150,000–800,000 Yuan	42.9	56.3	50.0	46.2
	Over 800,000 Yuan	25.0	22.9	28.6	23.1

Note: 100 Chinese Yuan = ~US$ 15.51 = ~€ 13.38 (as of 1 October 2021).

**Table 3:** Mann–Whitney *U* test for respondent perceptions between control and treatment groups.

Attribute	Respondent	Group	Count	Median	Mann-Whitney *U*	Significance (2-Tailed)
Label trust	Local	Control	28	3	544.5	0.1519
		Treatment	48	3		
	Foreign	Control	14	3	62.5	0.1556
		Treatment	13	4		
Meat quality	Local	Control	28	4	809	0.1152
		Treatment	48	4		
	Foreign	Control	14	5	97.5	0.7407
		Treatment	13	4		
Traceability trust	Local	Control	28	3	549.5	0.1783
		Treatment	48	3		
	Foreign	Control	14	3	59	0.1056
		Treatment	13	3		

**Table 4:** One-way ANOVA tests for treatment respondents’ characteristics on perception responses.

Dependent Variable	Respondent	Variable	Mean Square	*F* Value	Pr (>*F*)
Label trust	Local	City of residence	2.1451	2.245	0.0799 **
		Age	0.7806	0.716	0.615
		Education	1.363	1.325	0.276
		Gender and marital status	1.346	1.298	0.287
		Annual household income	1.337	1.298	0.286
	Foreign	City of residence	0.5534	0.286	0.834
		Age	5.276	14.61	0.00083 *
		Education	1.038	0.611	0.562
		Gender and marital status	1.228	0.693	0.617
		Annual household income	1.394	0.826	0.544
Meat quality	Local	City of residence	0.9422	1.033	0.401
		Age	1.0085	1.117	0.366
		Education	2.1219	2.645	0.0463 *
		Gender and marital status	0.5407	0.575	0.634
		Annual household income	1.2448	1.409	0.247
	Foreign	City of residence	0.2714	1.011	0.432
		Age	0.5103	2.701	0.108
		Education	0.1570	0.538	0.600
		Gender and marital status	0.2660	0.982	0.469
		Annual household income	0.1827	0.585	0.683
Traceability trust	Local	City of residence	3.836	2.743	0.0406 *
		Age	2.642	1.782	0.137
		Education	0.6125	0.361	0.835
		Gender and marital status	2.398	1.545	0.216
		Annual household income	2.291	1.485	0.223
	Foreign	City of residence	0.8397	1.778	0.221
		Age	1.0731	2.72	0.107
		Education	0.2596	0.415	0.671
		Gender and marital status	0.3173	0.462	0.763
		Annual household income	0.8173	1.868	0.210

Note: (a) * Significant level of 95%, ** Significant level of 90%; (b) Pr (>*F*) is the *p*-value associated with the F statistic of a given source.

**Table 5:** Respondent stated WTP more by group.

		Local Respondents		Foreign Respondents	
		Control	Treatment	Control	Treatment
Willingness to pay more	Yes	78.6%	64.6%	92.9%	84.6%
	No	-	4.2%	-	-
	Not sure	21.4%	31.3%	7.1%	15.4%
Min WTP more in RMB (Yuan)		3	2	10	10
Max WTP more in RMB (Yuan)		150	260	150	120
Mean WTP more in RMB (Yuan)		31.57	44.71	57.31	52.00
Standard deviation of WTP more		41.79	54.56	47.99	41.04

**Table 6:** Mann–Whitney *U* tests for WTP between control and treatment groups.

Attribute	Respondent	Group	Count	Median	Mann-Whitney *U*	Significance (2-Tailed)
WTP	Local	Control	28	110	819	0.1101
		Treatment	48	100		
	Foreign	Control	14	120	62.5	0.1556
		Treatment	13	120		
WTP more	Local	Control	21	20	261.5	0.09593
		Treatment	28	30		
	Foreign	Control	13	50	69.5	0.8024
		Treatment	10	50		

**Table 7:** One-way ANOVA tests for treatment respondents’ characteristics on WTP.

Dependent Variable	Respondent	Variable	Mean Square	*F* Value	Pr (>*F*)
WTP	Local	City of residence	1859	0.485	0.747
		Age	3036	0.811	0.548
		Education	2822	0.753	0.561
		Gender and marital status	8732	2.628	0.062 **
		Annual household income	6906	2.051	0.104
	Foreign	City of residence	113.0	0.129	0.941
		Age	1060.3	1.89	0.202
		Education	550.8	0.773	0.488
		Gender and marital status	442.1	0.547	0.707
		Annual household income	698.3	1.027	0.449
WTP more	Local	City of residence	6859	2.98	0.0404 *
		Age	3248	1.114	0.381
		Education	2156	0.7	0.561
		Gender and marital status	5470	2.053	0.133
		Annual household income	1889	0.607	0.617
	Foreign	City of residence	1114	0.566	0.657
		Age	2345	1.732	0.259
		Education	4090	4.102	0.0662 **
		Gender and marital status	1957	1.334	0.373
		Annual household income	707.5	0.287	0.875

Note: (a) * Significant level of 95%, ** Significant level of 90%; (b) Pr (>*F*) is the *p*-value associated with the F statistic of a given source.

## Appendix A

One-minute short video can be found online: https://v.qq.com/x/page/t3019jai4up.html (Chinese version) and https://youtu.be/Tec6L0xDqvE or https://v.qq.com/x/page/w3011zxg9ft.html (English version).

## Appendix B

The age divisions correspond with the socio-cultural context in China. Most university students in China are aged between 18 and 25, whereas people aged between 26 and 30 are generally those who are in their early career stage. People in their 30s, 40s and 50s are mature aged workers. The age groups have been set up accordingly.
